# Genomic analysis of carboxyl/cholinesterase genes in the silkworm *Bombyx mori*

**DOI:** 10.1186/1471-2164-11-377

**Published:** 2010-06-14

**Authors:** Takuya Tsubota, Takahiro Shiotsuki

**Affiliations:** 1 Invertebrate Gene Function Research Unit, National Institute of Agrobiological Sciences, 1-2 Owashi, Tsukuba, Ibaraki 305-8634, Japan

## Abstract

**Background:**

Carboxyl/cholinesterases (CCEs) have pivotal roles in dietary detoxification, pheromone or hormone degradation and neurodevelopment. The recent completion of genome projects in various insect species has led to the identification of multiple CCEs with unknown functions. Here, we analyzed the phylogeny, expression and genomic distribution of 69 putative CCEs in the silkworm, *Bombyx mori *(Lepidoptera: Bombycidae).

**Results:**

A phylogenetic tree of CCEs in *B. mori *and other lepidopteran species was constructed. The expression pattern of each *B. mori *CCE was also investigated by a search of an expressed sequence tag (EST) database, and the relationship between phylogeny and expression was analyzed. A large number of *B. mori *CCEs were identified from a midgut EST library. CCEs expressed in the midgut formed a cluster in the phylogenetic tree that included not only *B. mori *genes but also those of other lepidopteran species. The silkworm, and possibly also other lepidopteran species, has a large number of CCEs, and this might be a consequence of the large cluster of midgut CCEs. Investigation of intron-exon organization in *B. mori *CCEs revealed that their positions and splicing site phases were strongly conserved. Several *B. mori *CCEs, including juvenile hormone esterase, not only showed clustering in the phylogenetic tree but were also closely located on silkworm chromosomes. We investigated the phylogeny and microsynteny of neuroligins in detail, among many CCEs. Interestingly, we found the evolution of this gene appeared not to be conserved between *B. mori *and other insect orders.

**Conclusions:**

We analyzed 69 putative CCEs from *B. mori*. Comparison of these CCEs with other lepidopteran CCEs indicated that they had conserved expression and function in this insect order. The analyses showed that CCEs were unevenly distributed across the genome of *B. mori *and suggested that neuroligins may have a distinct evolutionary history from other insect order. It is possible that such an uneven genomic distribution and a unique neuroligin evolution are shared with other lepidopteran insects. Our genomic analysis has provided novel information on the CCEs of the silkworm, which will be of value to understanding the biology, physiology and evolution of insect CCEs.

## Background

The carboxyl/cholinesterase (CCE) superfamily is comprised of functionally diverse proteins that hydrolyze carboxylic esters to their component alcohols and acids. CCEs fall into three functional groups: dietary detoxification, hormone and pheromone degradation, and neurodevelopment [1,2].

The dietary detoxification group of CCEs includes esterases that are responsible for the metabolism of a broad range of substrates including xenobiotics in the diet and insecticides. There is evidence that the acquisition of insecticide resistance can arise either by mutations in CCE amino acid sequences that change the activity of the esterase or by amplification of CCE genes in this group [1]. Such phenomena have been observed in many insect species including flies, mosquitoes and aphids [1], and there might be common mechanisms for the acquisition of insecticide resistance in these species based on their CCEs. The hormone and pheromone degrading group includes juvenile hormone esterases (JHEs), pheromone degrading esterases (PDEs) and others. JHEs act to degrade juvenile hormone (JH), a sesquiterpenoid insect hormone that plays important roles in the regulation of a number of physiological processes [3-5]. The active functioning of JHE at the final instar larva is essential for normal larval-pupal metamorphosis [6]. PDEs are expressed in the adult male antenna and have a role in the degradation of sex pheromones produced by the female [7,8]. The degradation of the sex pheromone is believed to be essential to enable the male to accurately follow a pheromone trail. The third neurodevelopmental group includes acetylcholinesterases (AChEs), neuroligins, neurotactins, gliotactins and others. AChEs are the only CCEs of this group that are catalytically active and they function in neurotransmission [9]. With the exceptions of *Drosophila melanogaster *and other higher Diptera, insects have two AChE genes that show a clear 1:1 orthologous relationship between species [1]. Neuroligins are known to be involved in the cell-cell interactions of synapses [10]. The functions of neuroligins are well characterized in the human, mouse and rat [11,12], while recent studies in the honeybee, *Apis mellifera*, examined the splicing and expression of insect neuroligins [13] or revealed the genetic and functional conservation of neuroligins between vertebrate and invertebrate [14]. Not only neuroligins but also other CCEs in this group are catalytically inactive, as are some CCEs outside of the neurodevelopmental group, such as glutactins and β-esterases [1,15].

Recently, genome analyses have proceeded very rapidly in a wide range of species including insects. Insects were found to have multiple CCE genes, many of which have unknown function [1,2,16-19]. Determination of the functions of these genes based on sequence and homology information is infeasible. As members of the CCE superfamily have been found in prokaryotes to vertebrates, it is clear that elucidation of the roles of the genes in this family will have a wider biological relevance beyond entomology. With regard to genomic analyses, sequencing of the genome of the silkworm *Bombyx mori *has now been completed and released to public databases [20]. The silkworm is a useful model for lepidopteran insects, and comparative analyses between lepidopteran species can be made using the silkworm genomic information as a base. Moreover, the large body size of the silkworm has been exploited to establish multiple tissue-specific expressed sequence tag (EST) libraries [21,22]. Integration of genomic analysis and EST expression analysis should enable a more comprehensive understanding of the functions and evolution of many genes.

In this study, we used silkworm genomic information to analyze the phylogeny of lepidopteran CCEs. Based on a recent analysis of CCEs in the silkworm and *Helicoverpa armigera*, another species belonging to the Lepidoptera [23], we constructed a phylogenetic tree that included several novel lepidopteran CCEs. To gain further insight into the phylogeny of CCEs, we compared the expression patterns of each CCE by a search of an EST database. A large number of *B. mori *CCEs were identified in a midgut EST library and, interestingly, these were clustered in the phylogenetic tree. CCEs of other lepidopteran species that were positioned close to the cluster of *B. mori *midgut CCEs were also expressed in the midgut, suggesting that their functions are conserved between species. Additionally, we performed a comparative analysis of the intron-exon structure of *B. mori *CCE genes and determined their chromosomal locations. These analyses highlighted the unique phylogenetic character of *B. mori *neuroligins. Overall, our study has produced novel information on the CCEs of the silkworm and other lepidopteran insects, which will be of value to understanding the biology, physiology and evolution of insect CCEs.

## Results and Discussion

### B. mori CCEs

**Figure 1:**
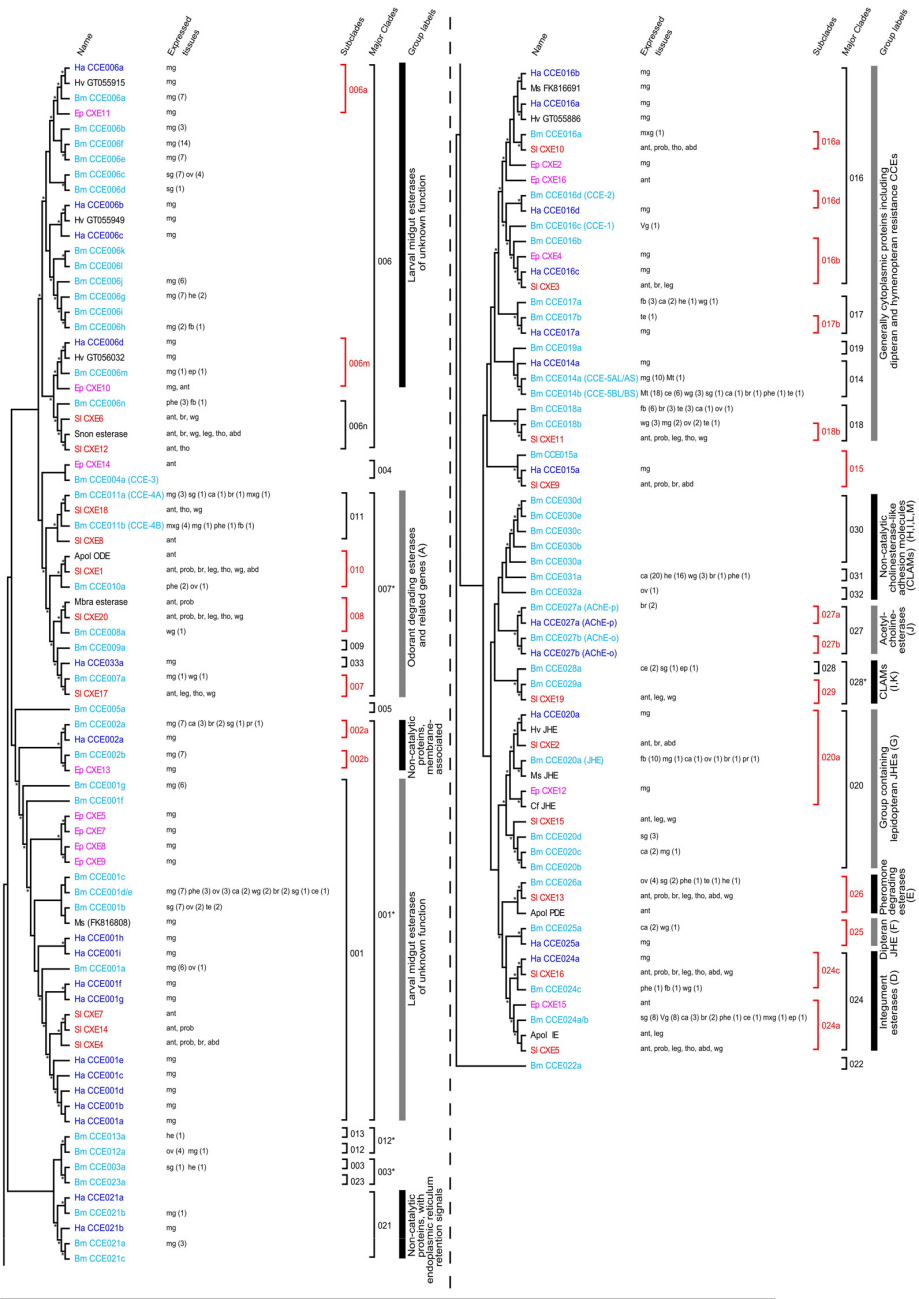
**Phylogenetic tree of CCEs**. MEGA 4.0 [40] was used to construct the phylogenetic tree using the Minimum Evolution method. Asterisks in the cladogram indicate bootstrap values greater than 50%. The nomenclatures of clades or groups are according to Teese et al [23]. Subclades of CCEs showing 1:1 orthologous relationship are marked red. The CCEs of *B. mori *are colored light blue, *H. armigera *blue, *S. littoralis *red and *E. postvittana *purple. The tissues in which each CCE is expressed are shown to the right of CCE name. For *B. mori *CCEs, the number of EST clones identified by the database analysis is shown in parenthesis. Species name abbreviations: Ha, *Helicoverpa armigera*; Hv, *Heliothis virescens*; Bm, *Bombyx mori*; Ep, *Epiphyas postvittana*; Sl, *Spodoptera littoralis*; Snon, *Sesamia nonagrioides*; Apol, *Antheraea polyphemus*; Mbra, *Mamestra brassicae*; Ms, *Manduca sexta*; Cf, *Choristoneura fumiferana*. Abbreviations for tissues: mg, midgut; sg, silk gland; ov, ovary; he, hemocyte; fb, fat body; ep, epidermis; ant, antenna; phe, pheromone gland; br, brain; wg, wing; tho, thorax; abd, abdomen; ca, corpora allata; mxg, maxillary galea; prob, proboscis; pr, prothoracic gland; ce, compound eye; te, testis; Vg, Verson's gland; Mt, Malpighian tubules.

A recent study identified 70 putative CCEs in *B. mori *[23]. Our present study is largely in accordance with that work, including the following minor exceptions. In our analysis, BmCCE001d and 001e was dealt with as a single gene because they have slight differences in amino acid sequence, and a search of KAIKObase identified only one genomic locus corresponding to them [20]; this is also the case for BmCCE024a and 024b. On the other hand, BGIBMGA002185 was included among putative CCEs as our phylogenetic analysis placed this gene in the same cluster as BmCCE030d with a bootstrap value of more than 50% (Figure 1). Using the nomenclature system proposed by Teese et al [23], this CCE was designated BmCCE030e (Figure 1). In total, we focused on 69 *B. mori *CCEs in this study.

### Construction of the phylogenetic tree of lepidopteran CCEs

A phylogenetic tree of lepidopteran CCEs is shown in Figure 1. This tree contains CCEs of *B. mori*, *H. armigera *and several other lepidopteran species (see Figure 1); the CCEs of *Spodoptra littoralis*, *Heliothis virescens *and *Manduca sexta *have only recently been identified [24,25]. Comparison of the relationship between *B. mori *and other lepidopteran CCEs revealed that among 69 *B. mori *CCEs 21 appeared to have a 1:1 orthologous relationship with CCEs of other lepidopteran species, while others not (Figure 1).

Although Teese et al [23] proposed 33 major clades for insect CCEs, the phylogenetic tree produced here after inclusion of additional CCEs suggested that several of these clades could be merged. The integration of clades 001 and 002 as clade 001*, clades 003 and 023 as clade 003*, clades 012 and 013 as clade 012*, clades 028 and 029 as clade 028*, and clades 007-011 and 033 as clade 007* was supported with a bootstrap value of greater than 50% (Figure 1).

One striking characteristic of *B. mori *is the presence of a much greater number of CCEs compared to species in other insect orders [23]. Analysis of CCE phylogeny has shown that a large cluster containing clades 001*, 004-007* and others are Lepidoptera-specific (Additional file 1). These clades contain more than 30 *B. mori *CCEs (Figure 1, Additional file 1)). CCE clusters specific to non-lepidopteran orders have also been identified [2,18,19]; however, none of these clusters contains as many CCEs as those in the Lepidoptera for *B. mori*. This suggests that the abundance of CCEs in *B. mori *(and possibly in other lepidopteran species) is related to the existence of this large Lepidoptera-specific cluster.

### EST clone analysis of B. mori CCEs

To further investigate the functions of *B. mori *CCEs and the relationships between CCE phylogeny and expression profile, we searched a silkworm EST database to identify the tissues in which each CCE was expressed. In total, the search found 354 EST clones with homology to CCE in libraries from larval or pupal tissues; these clones corresponded to 47 CCE genes (Tables 1 and 2). A summary of the expression patterns of the CCEs is given in Figure 1. Recently, we described the developmental expression profiles of several CCE genes [26]. The EST database search here showed good agreement with the results of this earlier analysis of developmental expression patterns. Thus, for example, multiple clones of JHE were found in the fat body library (Figure 1), consistent with the high level of expression of *jhe *in the fat body of the final instar stage larva [26,27]. Similarly, EST clones of CCE011a/b (CCE-4A/B) were present in a range of tissues (Figure 1) and were previously shown to be expressed in these tissues [26]. Such consistency was also obtained for CCE014a (CCE-5AL/AS) and CCE014b (CCE-5BL/BS) (Figure 1, [26]), further supporting the validity of our EST expression analysis.

**Table 1:** Frequency distribution of *B. mori *CCE genes based on the number of EST clones identified by the database search.

Number of EST clones	CCE gene
0	22
1	8
2	4
3	8
4	3
5	1
6	2
7	7
8	1
9	2
10	0
11	3
12	0
13	0
14	3
>15	5

**Table 2:** Frequency distribution of *B. mori *CCE genes based on the tissues from which the EST clones were derived.

tissue	CCE gene	Number of EST clones
midgut	23	104
corpora allata	12	39
silk gland	12	34
ovary	12	25
brain	10	16
pheromone gland	10	15
wing	9	16
fat body	8	25
hemocyte	6	22
testis	5	8
compound eyes	4	10
maxillary galea	4	7
epidermis	3	3
Malpighian tubules	2	19
Verson's gland	2	9
prothoracic gland	2	2

The largest group of EST clones was identified in the larval midgut library: 104 of the total 354 clones came from this library, and they corresponded to 23 CCEs (Figure 1, Table 2). The majority of the midgut CCEs belonged to lepidoptera-specific phylogenetic clades (Figure 1, Additional file 1)), suggesting that the large number of silkworm CCEs (and possibly of other lepidopteran species) might be a consequence of this large number of midgut CCEs. Overall, however, *B. mori *had slightly fewer midgut CCEs than *H. armigera *[23]. This might reflect differences in feeding behavior of the two species: *B. mori *is monophagous, while *H. armigera *is polyphagous. In addition to the midgut, the analysis of the EST cDNA libraries showed expression of CCEs in the corpora allata, silk gland, ovary, brain, pheromone gland, wing, fat body, hemocyte, and testis (Table 2). In *D. melanogaster *species subgroup, it is known that a CCE expressed in the male ejaculatory duct is transferred to the female via the semen during mating and that this CCE stimulates egg laying behavior and inhibits the receptivity to remating in the female [28]. It is possible that *B. mori *CCEs expressed in the male testis have similar functions although the precise expression pattern might be different. However, in most cases, the functions of CCEs in each tissue are unknown.

### Relationship between CCE expression profile and phylogeny

We sought to determine if there was any relationship between CCE phylogeny and patterns of expression in tissues. Many of the CCEs in clade 001* were confirmed to be expressed in the midgut (Figure 1). Although the CCEs of *S. littoralis *in this clade were derived from an antennal EST library [24], it might be possible that they are also expressed in the larval midgut. CCEs of subclade 001 are considered to be catalytically active, and one of their possible roles is the detoxification of noxious substances in the diet. By contrast, CCEs of subclade 002 lack the catalytic serine residue and are presumed to be inactive, although they might bind to substrates in the midgut. Expression of catalytically inactive CCEs of clade 021 was also found in the midgut (Figure 1).

Many of the *B. mori *CCEs in clade 006 were expressed in the midgut (Figure 1). Likewise, CCEs of clade 006 from several other insect species are also expressed in the midgut (Figure 1, [23,25,29]). On the basis of these results, we named clade 006 "larval midgut esterases of unknown function", a designation different from that used by Teese et al [23]. It should be noted that BmCCE006c and 006d are mainly expressed in the silk gland, suggesting that novel CCEs closely related to these silk gland proteins might be identified in other lepidopteran species in the future. As no clone of BmCCE006n was found in the midgut library, and the other CCEs of subclade 006n originated from the antenna, we tentatively excluded this subclade from "larval midgut esterases of unknown function" (Figure 1).

In contrast to the CCEs described above, those in clade 007* were derived from various tissues (Figure 1). Subclades 008 and 010 included CCEs from antenna [24,30,31]. Currently, it is not known whether BmCCE008a and BmCCE010a are expressed in the antenna; nevertheless, it is still possible that subclades 008 and 010 form an antennal CCE cluster. By contrast, BmCCE011a/b are expressed in various organs (see above). Thus, CCEs in this cluster might have a universal function rather than a tissue-specific role. BmCCE011a and 011b have been shown to be alternative splicing products of the same gene and to share a 62 amino acid sequence at their N-termini [26]. Interestingly, SlCXE8 and SlCXE18 also have a common 62 amino acid sequence at their N-termini, indicating that such alternative splicing might be conserved among lepidopteran species.

Among the CCEs of clade 014, BmCCE014a and 014b are also splicing variants of the same gene [26]. BmCCE014a is expressed strongly in the midgut and Malpighian tubules, and this gene showed strong activity for degrading 1-naphthyl acetate (1-NA), a general esterase substrate [26]. Interestingly, the *H. armigera *homologue, HaCCE014a, is also expressed in the midgut and also has the ability to degrade 1-NA [23], suggesting that not only expression but also function of CCEs in this clade is conserved between species.

Four *B. mori *CCEs are located in clade 016 (Figure 1); none were confirmed to be expressed in the midgut. This outcome is consistent with a previous analysis of the expression profile of BmCCE016c (CCE-1) and BmCCE016 d (CCE-2) [26]. Other insect species, however, have homologous CCEs that are expressed in the midgut (Figure 1). Thus, the expression patterns of CCEs in this cluster might not be conserved among species.

CCEs of clades 018, 024 and 026 appear to be expressed ubiquitously (Figure 1), suggesting they might have universal roles, in a similar manner to CCEs of subclade 011. One exception is *Antheraea polyphemus *PDE of clade 026, which is specifically expressed in the adult male antenna [7]. In contrast, the *B. mori *homologue, BmCCE026a, is expressed in various tissues (Figure 1). This may reflect functional differences between these CCEs, possibly related to species differences with respect to usage of sex pheromones. The sex pheromones of *A. polyphemus *are ester compounds while those of *B. mori *are a mixture of an alcohol and an aldehyde. However, *S. littoralis *is also known to use ester compounds as sex pheromones, but SlCXE13, the putative counterpart to *A. polyphemus *PDE, surprisingly shows ubiquitous expression [24]. One possible explanation is that the *A. polyphemus *PDE has a specified function for the degradation of the sex pheromone, while SlCXE13 has functions in addition to pheromone degradation.

### Intron-exon organization

**Figure 2:**
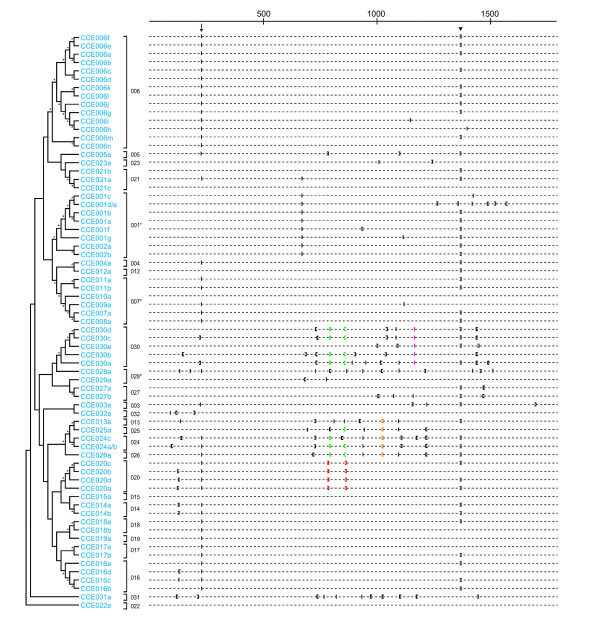
**Phylogenetic tree and intron positions of silkworm CCE genes**. Asterisks in the cladogram indicate bootstrap values greater than 50%, and the nomenclatures of clades are according to Teese et al [23]. The intron position of sequences is shown as (|) for a phase 0 intron, ([) for a phase 1 intron and (]) for a phase 2 intron. The arrow indicates the phase 0 intron at position 229 or 230, while the arrowhead shows the phase 2 intron at position 1368. The green brackets indicate the phase 1 introns at positions 792 and 861 shared among CCEs in clades 024-026 and 030, orange brackets the phase 1 intron at position 1022 shared among CCEs in clades 013 and 024-026, purple bars the phase 0 intron at position 1165 shared among CCEs in clade 030 and red brackets the phase 2 introns at position 787 and 865 shared among CCEs in clade 020.

**Figure 3:**
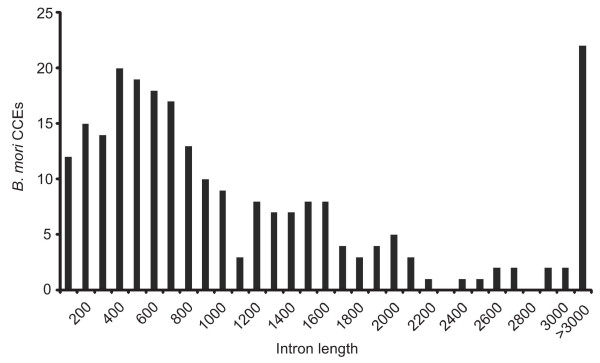
**The distribution of the intron length in *B. mori *CCEs**.

Next, we investigated the intron-exon organization of *B. mori *CCEs. In total, 240 introns were identified in the *B. mori *CCEs. Four CCEs were intronless (Figure 2), the remainder had one to thirteen introns each (Figure 2). The average intron size was 1372 nucleotides. The longest intron was present in BmCCE027b and comprised 13962 nucleotides located between exons 2 and 3. BmCCE020c, BmCCE020d and BmCCE025a contained the shortest introns of 68 nucleotides. Such intron size variations are similarly observed in *B. mori *glutathione-S-transferases (GST) [32]. The intron size distribution in *B. mori *CCEs is shown in Figure 3. The lengths of the introns showed an approximately even distribution.

We mapped the positions of introns in *B. mori *CCEs by the multiple sequence alignment (Figure 2). There was a clear and strong conservation of intron positions among the CCEs, as was also observed for *B. mori *GSTs (Figure 2, [32]). We also classified the splice sites into three phases according to their positions in the codons: phase 0 for a splice site lying between two codons, phase 1 for a splice site lying one base inside a codon in the 3' direction, and phase 2 for a splice site lying two bases inside the codon in the 3' direction. We then examined the distribution of these three splice site phases and found that not only the position of the intron but also the splice site phase was strongly conserved (Figure 2). The most conserved intron was a phase 2 intron at position 1368; this was present in 45 CCEs (Figure 2, arrowhead). A phase 0 intron at position 229 or 230 was also present in 20 CCEs, respectively (Figure 2, arrow). Fifty-seven *B. mori *CCEs contained one or both of these introns (Figure 2), indicating that these arose at an early stage of CCE evolution. In addition to these two introns, others were also conserved in several clades. Phase 2 introns at positions 787 and 865 were conserved in all CCEs of clade 020 (Figure 2, red brackets), a phase 1 intron at position 1022 was present in 5 CCEs of clade 013 and 024-026 (Figure 2, orange brackets), and a phase 0 intron at position 1165 was present in all CCEs of clade 030 (Figure 2, purple bars). On the other hand, 3 intron positions are conserved in all CCEs of clade 20, and 4 introns are conserved in 3 CCEs of this clade (Figure 2). Such a clade-specific strong conservation of intron phase and position was also observed for *B. mori *GSTs [32]. Interestingly, CCEs of clades 024-026 and 030 had a phase 1 intron at positions 792 and 861 (Figure 2, green brackets), despite their distant locations in the phylogenetic tree (Figures. 1 and 2). As described below, these two introns were also conserved in the neuroligins of *D. melanogaster *and *A. mellifera*. Totally, we found 21 intron positions that are conserved in more than 2 *B. mori *CCEs.

### Chromosomal locations of CCEs in the silkworm

**Figure 4:**
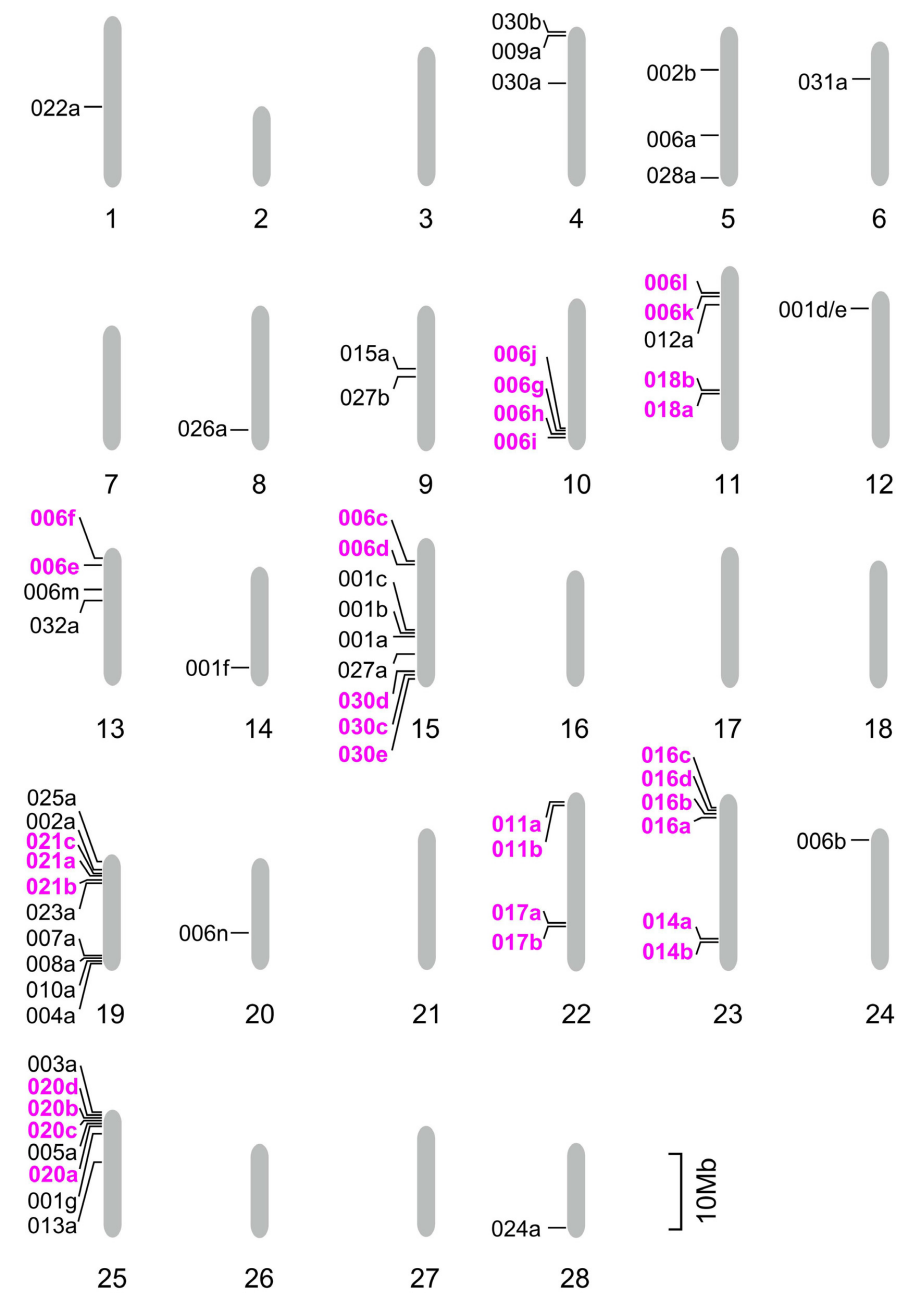
**Chromosomal locations of silkworm CCE genes**. The length of each chromosome is drawn to scale. CCEs that are clustered on chromosomes and the phylogenetic tree (Figures. 1 and 2) are colored purple. CCE019a, 024c and 029a were located in a scaffold whose chromosomal location is unknown and is not shown on this figure.

**Figure 5:**
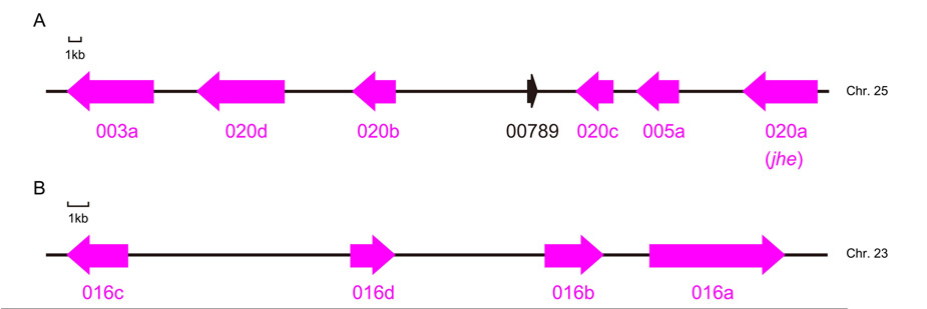
**The organization of the CCE cluster around CCE020 and 016 in the silkworm genome**. Six CCEs are clustered around CCE020a on chromosome 25, along with one additional gene (BGIBMGA000789) (A); four CCEs are in a cluster on chromosome 23 (B). The arrows indicate the direction of transcription. CCEs are shown as purple arrows, while other genes are indicated with black arrows.

Examination of the chromosomal locations of silkworm CCEs showed these were distributed unevenly across the genome (Figure 4). A more detailed representation of the genomic structure of the clusters on chromosomes 25 and 23 is shown in Figure 5. Six CCEs on chromosome 25 are in the same orientation, while four CCEs on chromosome 23 vary in orientation (Figures. 4 and 5). This clustered distribution pattern has also been observed for silkworm GSTs [32]. The chromosomal clusters of CCEs of clades 016 and 020 indicate that they arose through a recent duplication. In addition to these clades, other CCEs showed clustering on the silkworm genome and, in many cases, also showed clustering in the phylogenetic tree (Figures. 1, 2 and 4). A recent study found evidence of conserved microsynteny in Lepidoptera [33,34]. It is possible that a similar phenomenon occurs with regard to CCE chromosomal clusters in other lepidopteran insects.

Genomic clustering of CCEs has also been observed for non-lepidopteran insects such as *D. melanogaster *and *Nasonia vitripennis *[1,18]. In *D. melanogaster *a large CCE cluster has been identified on chromosome 3R [1]; however, neither *B. mori *nor *N. vitripennis *have such a large CCE cluster (Figure 4, [18]). On the other hand, there are several differences between the chromosomal locations of CCEs in *B. mori *and *N. vitripennis*. CCE clusters in *N. vitripennis *tend to be localized around centromeric regions [18], whereas in *B. mori*, the clusters were frequently observed close to the telomeric regions (Figure 4). Another difference is that while the three functional classes of CCEs are respectively clustered in the chromosomes of *N. vitripennis *[18], no such functional clustering was observed in *B. mori *CCEs (Figure 4).

We also analyzed the relationship between the chromosomal location of *B. mori *CCEs and the tissues in which they were expressed. In some cases, adjacently located CCEs were expressed in the same tissue; for example, CCE006g, 006h and 006j were expressed in the midgut, and CCE006c and 006d were expressed in the silk gland (Figures. 1 and 4). This might indicate that these CCEs were born via a recent duplication event. However, CCEs located adjacently are not always expressed in the same tissue; such CCEs are probably regulated by independent enhancers/promoters that have distinct activities, despite their close chromosomal locations. Disagreement between chromosomal location and expression pattern has also been reported for silkworm cuticular protein genes [35]. Such CCEs might have distinct functions in the silkworm.

### Analysis of phylogeny and microsynteny in neuroligin genes

Finally, we investigated the phylogeny and chromosomal locations of neuroligins, genes in clade 30 in the phylogenetic tree, in more detail (Figure 1). Every insect genome examined to date contains multiple neuroligin-like sequences, and phylogenetic analyses have indicated that these sequences are highly conserved [2,13]. Moreover, it was also reported that vertebrate and invertebrate neuroligins are conserved genetically and functionally [14].

**Figure 6:**
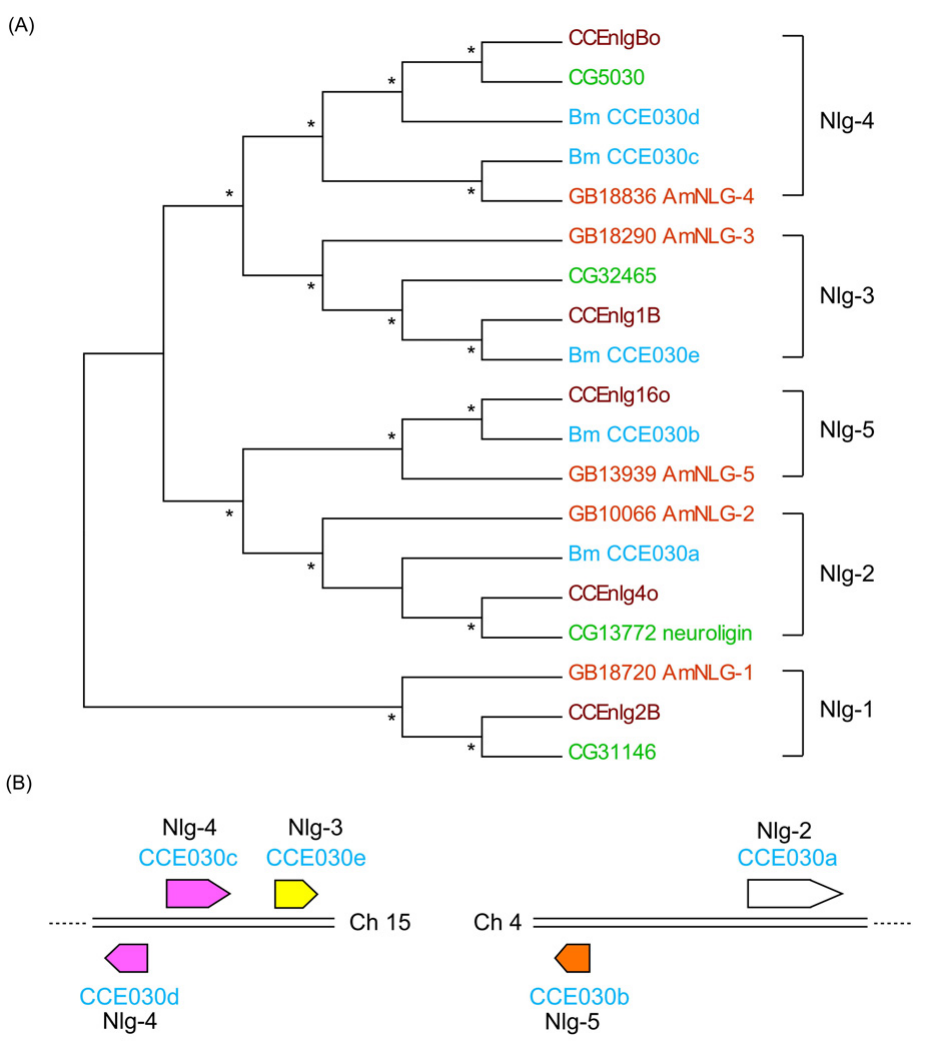
**Phylogenetic and microsynteny analysis of *neuroligin *genes**. (A) The phylogenetic tree of *B. mori*, *D. melanogaster*, *A. aegypti *and *A. mellifera neuroligin*s. Asterisks in the cladogram indicate bootstrap values greater than 50%. For the neuroligins except *B. mori*, refer to Claudianos et al [2]. Neuroligins of *B. mori *are colored light blue, *D. melanogaster *are colored green, *A. gambiae *are colored brown and *A. mellifera *are colored orange. (B) The chromosomal locations of *B. mori *neuroligins. The direction of the arrow indicates the transcription direction. The color of each neuroligin arrow is the same as that for orthologs in Claudianos et al [2].

Our analysis of the *B. mori *genome identified five putative neuroligins, CCE030a-e (Figures. 1 and 2). We constructed another phylogenetic tree using the sequence data for the five *B. mori *neuroligins, four *D. melanogaster *neuroligins, five *A. gambiae *neuroligins and five *A. mellifera *neuroligins (Figure 6A). Each species had at most one CCE in each neuroligin subcluster, while *B. mori *had two CCEs in the Nlg-4 subcluster (Figure 6A), the first evidence of a neuroligin duplication event in the Insecta. Although a CCE corresponding to Nlg-1 could not be identified in *B. mori*, BGIBMGA002170, which showed very weak homology to other insect Nlg-1s, is a candidate homologue. BGIBMGA002170 was not located in the same clade as other neuroligins in the phylogenetic tree (data not shown); however, our microsynteny analysis supported the interpretation of Nlg-1 homology (see below).

The chromosomal locations of *B. mori *neuroligins showed unique features compared to other insects (Figure 6B). In *A. mellifera*, *A. gambiae *and *D. melanogaster*, all neuroligins except for Nlg-2 are located on the same chromosome [2]. By contrast, *B. mori *Nlg-3 and Nlg-4 are located on a chromosome different from the one Nlg-5 is located; Nlg-3 and two Nlg-4 genes are on chromosome 15, while Nlg-5 is on chromosome 4 (Figures. 4 and 6B). Moreover, Nlg-2, which is singly located on a chromosome in *D. melanogaster*, *A. gambiae *and *A. mellifera*, is on the same chromosome as Nlg-5 in *B. mori *(Figure 6B). BGIBMGA002170 is located between Nlg-3 and two Nlg-4 genes (data not shown). In *D. melanogaster*, *A. gambiae *and *A. mellifera *Nlg-1 is located between Nlg-3 and Nlg-4 [2], supporting for the interpretation that BGIBMGA002170 is an Nlg-1 homologue. In light of these results, we propose that the following events have occurred in the evolution of the Lepidoptera: (1) duplication of the Nlg-4 gene, (2) separation of the chromosomal segments containing Nlg-3~5, and (3) fusion of the chromosomal segments containing Nlg-2 and Nlg-5. More genomic information will be necessary to verify this hypothesis. We also compared the intron positions between neuroligins of *B. mori*, *D. melanogaster *and *A. mellifera*, and found that most intron positions, including the common intron in CCEs of clades 024-026 (Figure 2, green brackets), were conserved.

## Conclusions

We analyzed the genomic distribution, phylogeny and EST expression of 69 *B. mori *CCEs. Many *B. mori *CCEs were expressed in the midgut, and such midgut expression was conserved with CCEs of other lepidopteran insects located in the same phylogenetic tree. The abundance of CCEs of the silkworm (or Lepidoptera), compared to species of other insect orders, is a possible consequence of the high number of these midgut CCEs. Intron positions and splice site phases were strongly conserved among *B. mori *CCEs, and they were located unevenly in the genome. Among the CCEs of *B. mori*, neuroligins show evidence of having evolved uniquely compared to other insects. Our genomic analysis has provided novel information on the CCEs of the silkworm, which will be of value to understanding the biology, physiology and evolution of insect CCEs.

## Methods

### Database analysis

CCE sequences were retrieved from NCBI [36]. EST clones of *B. mori *CCEs were searched using tBLASTN in NCBI [36], KAIKObase [37], SilkBase [38] and a private library. Introns were identified by comparison of amino acid sequences with DNA sequences, and the canonical GT/AG rule was used to specify the exon-intron junction position [39]. The chromosomal locations of the genes were determined from KAIKObase [37].

### Phylogenetic analysis of CCE

ClustalW software was used to perform a multiple sequence alignment prior to the phylogenetic analysis. MEGA 4.0 [40] was used to construct the phylogenetic tree using the Minimum Evolution method with the JTT matrix. To evaluate branch strength in the phylogenetic tree, a bootstrap analysis of 500 replicates was performed.

## Abbreviations

CCE: carboxyl/cholinesterase; JHE: juvenile hormone esterase; GST: glutathione-S-transferase; EST: expressed sequence tag; 1-NA: 1-naphthyl acetate; NCBI: National Center for Biotechnology Information

## Authors' contributions

TT carried out all of the analysis described in this paper and wrote the manuscript. TS completed the manuscript. All authors read and approved the final manuscript.

## Supplementary Material

Additional file 1 **Phylogenetic tree of insect CCEs**. A phylogenetic tree containing lepidopteran, *D. melanogaster*, *A. mellifera*, *T. castaneum *and *A. pisum *CCEs. Lepidopteran CCEs are colored blue, *D. melanogaster *purple, *A. mellifera *orange, *T. castaneum *brown and *A. pisum *green. Asterisks in the cladogram indicate bootstrap values greater than 50%, and the nomenclatures of clades are according to Teese et al [23]. For simplicity, neurodevelopmental CCEs are omitted. Click here for file

## References

[B1] Oakeshott JG Claudianos C Campbell PM Newcomb RD Russell RJ Gilbert LI, Iatrou K, Gill SS Biochemical genetics and genomics of insect esterases Comprehensive Molecular Insect Science-Pharmacology 2005 5 Oxford: Elsevier 309 381 full_text

[B2] Claudianos C Ranson H Johnson RM Biswas S Schuler MA Berenbaum MR Feyereisen R Oakeshott JG A deficit of detoxification enzymes: pesticide sensitivity and environmental response in the honeybee Insect Mol Biol 2006 15 615 636 10.1111/j.1365-2583.2006.00672.x 17069637 PMC1761136

[B3] Riddiford LM Cellular and molecular actions of juvenile hormone 1. General considerations and premetamorphic actions Advances in Insect Physiology 1994 24 213 274 full_text

[B4] Wyatt GR Davey KG Cellular and molecular actions of juvenile hormone 2. Roles of juvenile hormone in adult insects Advances in Insect Physiology 1996 26 1 155 full_text

[B5] Jallon JM Wicker-Thomas C Blomquist GJ, Vogt RG Genetic studies on pheromone production in Drosophila Insect Pheromone Biochemistry and Molecular Biology 2003 Oxford: Elsevier 253 281 full_text

[B6] Prestwich GD Eng WS Roe RM Hammock BD Synthesis and bioassay of isoprenoid 3-alkylthio-1,1,1-trifluoro-2-propanones: potent, selective inhibitors of juvenile hormone esterase Arch Biochem Biophys 1984 228 639 645 10.1016/0003-9861(84)90033-X 6696451

[B7] Ishida Y Leal WS Rapid inactivation of a moth pheromone Proceedings of the National Academy of Sciences of the United States of America 2005 102 14075 14079 10.1073/pnas.0505340102 16172410 PMC1216831

[B8] Ishida Y Leal WS Chiral discrimination of the Japanese beetle sex pheromone and a behavioral antagonist by a pheromone-degrading enzyme Proceedings of the National Academy of Sciences of the United States of America 2008 105 9076 9080 10.1073/pnas.0802610105 18579770 PMC2440356

[B9] Massoulie J Sussman J Bon S Silman I Structure and functions of acetylcholinesterase and butyrylcholinesterase Prog Brain Res 1993 98 139 146 full_text 8248501 10.1016/s0079-6123(08)62391-2

[B10] Song JY Ichtchenko K Sudhof TC Brose N Neuroligin 1 is a postsynaptic cell-adhesion molecule of excitatory synapses Proceedings of the National Academy of Sciences of the United States of America 1999 96 1100 1105 10.1073/pnas.96.3.1100 9927700 PMC15357

[B11] Ichtchenko K Hata Y Nguyen T Ullrich B Missler M Moomaw C Sudhof TC Neuroligin 1: a splice site-specific ligand for β-neurexins. Cell 1995 81 435 443 10.1016/0092-8674(95)90396-8 7736595

[B12] Bolliger MF Frei K Winterhalter KH Gloor SM Identification of a novel neuroligin in humans which binds to PSD-95 and has a widespread expression. Biochemical J 2001 356 581 588 10.1042/0264-6021:3560581 PMC1221872 11368788

[B13] Biswas S Russell RJ Jackson CJ Vidovic M Ganeshina O Oakeshott JG Claudianos C Bridging the synaptic gap: neuroligins and neurexin I in Apis mellifera. Plos one 2008 3 e3542 10.1371/journal.pone.0003542 18974885 PMC2570956

[B14] Biswas S Reinhard J Oakeshott J Russell R Srinivasan MV Claudianos C Sensory regulation of *neuroligins *and *neurexin I *in the honeybee brain Plos one 2010 5 e9133 10.1371/journal.pone.0009133 20161754 PMC2817746

[B15] Robin C Bardsley LMJ Coppin C Oakeshott JG Birth and death of genes and functions in the β-esterase cluster of *Drosophila* J Mol Evol 2009 69 10 21 10.1007/s00239-009-9236-3 19536450 PMC2706376

[B16] Ranson H Claudianos C Ortelli F Abgrall C Hemingway J Sharakhova MV Unger MF Collins FH Feyereisen R Evolution of supergene families associated with insecticide resistance Science 2002 298 179 181 10.1126/science.1076781 12364796

[B17] Strode C Wondji CS David JP Hawkes NJ Lumjuan N Nelson DR Drane DR Karunaratne SHPP Hemingway J Black IV WC Ranson H Genomic analysis of detoxification genes in the mosquito Aedes aegypti Insect Biochem Mol Biol 2008 38 113 123 10.1016/j.ibmb.2007.09.007 18070670

[B18] Oakeshott JG Johnson RM Berenbaum MR Ranson H Cristino AS Claudianos C Metabolic enzymes associated with xenobiotic and chemosensory responses in *Nasonia vitripennis* Insect Mol Biol 2010 19 Suppl 1 147 163 10.1111/j.1365-2583.2009.00961.x 20167025

[B19] Ramsey JS Rider DS Walsh TK De Vos M Ponnala L Macmil SL Roe BA Jander G Comparative analysis of detoxification enzymes in *Acyrthosiphon pisum *and *Myzus persicae* Insect Mol Biol 2010 19 Suppl 2 155 164 10.1111/j.1365-2583.2009.00973.x 20482647

[B20] The International Silkworm Genome Consortium The genome of a lepidopteran model insect, the silkworm Bombyx mori. Insect Biochem Mol Biol 2008 38 1036 1045 10.1016/j.ibmb.2008.11.004 19121390

[B21] Mita K Morimyo M Okano K Koike Y Nohata J Kawasaki H Kadono-Okuda K Yamamoto K Suzuki MG Shimada T Goldsmith MR Maeda S The construction of an EST database for *Bombyx mori *and its application Proceedings of the National Academy of Sciences of the United States of America 2003 100 14121 14126 10.1073/pnas.2234984100 14614147 PMC283556

[B22] Xia Q Zhou Z Lu C Cheng D Dai F Li B Zhao P Zha X Cheng T Chai C Pan G A draft sequence for the genome of the domesticated silkworm (*Bombyx mori*) Science 2004 306 1937 1940 10.1126/science.1102210 15591204

[B23] Teese MG Campbell PM Scott C Gordon KHJ Southon A Hovan D Robin C Russell RJ Oakeshott JG Gene identification and proteomic analysis of the esterases of the cotton bollworm, *Helicoverpa armigera* Insect Biochem Mol Biol 2010 40 1 16 10.1016/j.ibmb.2009.12.002 20005949

[B24] Durand N Carot-Sans G Chertemps T Montagné N Jacquin-Joly E Debernard S Maïbèche-Coisne M A diversity of putative carboxylesterases are expressed in the antennae of the noctuid moth *Spodoptera littoralis* Insect Mol Biol 2010 19 87 97 10.1111/j.1365-2583.2009.00939.x 20002215

[B25] Govind G Mittapalli O Griebel T Allmann S Böcker S Baldwin IT Unbiased transcriptional comparisons of generalist and specialist herbivores feeding on progressively defenseless *Nicotiana attenuate *plants PLoS One 2010 5 e8735 10.1371/journal.pone.0008735 20090945 PMC2806910

[B26] Tsubota T Shimomura M Ogura T Seino A Nakakura T Mita K Shinoda T Shiotsuki T Molecular characterization and functional analysis of novel carboxyl/cholinesterases with GQSAG motif in the silkworm *Bombyx mori* Insect Biochem Mol Biol 2010 40 100 112 10.1016/j.ibmb.2009.12.015 20060470

[B27] Hirai M Kamimura M Kikuchi K Yasukochi Y Kiuchi M Shinoda T Shiotsuki T cDNA cloning and characterization of *Bombyx mori *juvenile hormone esterase: an inducible gene by the imidazole insect growth regulator KK-42 Insect Biochem Mol Biol 2002 32 627 635 10.1016/S0965-1748(01)00141-2 12020837

[B28] Richmond RC Nielsen KM Brady JP Snella EM Barker JSF, Starmer WT, MacIntyre RJ Physiology, biochemistry and molecular biology of the *Est*-*6 *locus in *Drosophila melanogaster* Ecological and Evolutionary Genetics of Drosophila 1990 New York: Plenum 273 293

[B29] Simpson RM Newcomb RD Gatehouse HS Crowhurst RN Chagné D Gatehouse LN Markwick NP Beuning LL Murray C Marshall SD Yauk Y-K Nain B Wang Y-Y Gleave AP Christeller JT Expressed sequence tags from the midgut of *Epiphyas postvittana *(Walker) (Lepidoptera: Tortricidae) Insect Mol Biol 2007 16 675 690 18092997 10.1111/j.1365-2583.2007.00763.x

[B30] Ishida Y Leal WS Cloning of putative odorant-degrading enzyme and integumental esterase cDNAs from the wild silkmoth, *Antheraea polyphemus* Insect Biochem Mol Biol 2002 32 1775 1780 10.1016/S0965-1748(02)00136-4 12429129

[B31] Maïbèche-Coisne M Merlin C François M-C Queguiner I Porcheron P Jacquin-Joly E Putative odorant-degrading esterase cDNA from the moth *Mamestra brassicae*: cloning and expression patterns in male and female antennae Chem Senses 2004 29 381 390 10.1093/chemse/bjh039 15201205

[B32] Yu Q Lu C Li B Fang S Zuo W Dai F Zhang Z Xiang Z Identification, genomic organization and expression pattern of glutathione S-transferase in the silkworm, *Bombyx mori*. Insect Biochem Mol Biol 2008 38 1158 1164 10.1016/j.ibmb.2008.08.002 19280710

[B33] Grubor VD Heckel DG Evaluation of the role of CYP6B cytochrome P450 s in pyrethroid resistant Australian *Helicoverpa armigera* Insect Mol Biol 2007 16 15 23 10.1111/j.1365-2583.2006.00697.x 17257205

[B34] d'Alençon E Sezutsu H Legeai F Permal E Bernard-Samain S Gimenez S Gagneur C Cousserans F Shimomura M Brun-Barale A Flutre T Couloux A East P Gordon K Mita K Quesneville H Fournier P Feyereisen R Extensive synteny conservation of holocentric chromosomes in Lepidoptera despite high rates of local genome rearrangements Proceedings of the National Academy of Sciences of the United States of America in press 10.1073/pnas.0910413107 PMC2867904 20388903

[B35] Futahashi R Okamoto S Kawasaki H Zhong Y Iwanaga M Mita K Fujiwara H Genome-wide identification of cuticular protein genes in the silkworm, Bombyx mori Insect Biochem Mol Biol 2008 38 1138 1146 10.1016/j.ibmb.2008.05.007 19280704

[B36] NCBI http://www.ncbi.nlm.nih.gov/

[B37] KAIKObase http://sgp.dna.affrc.go.jp/KAIKObase/

[B38] SilkBase http://silkbase.ab.a.u-tokyo.ac.jp/cgi-bin/index.cgi

[B39] Mount SM A catalogue of splice junction sequences Nucleic Acids Res 1982 10 459 472 10.1093/nar/10.2.459 7063411 PMC326150

[B40] Tamura K Dudley J Nei M Kumar S MEGA4: Molecular Evolutionary Genetics Analysis (MEGA) software version 4.0 Mol Biol Evol 2007 24 1596 1599 10.1093/molbev/msm092 17488738

