# Supplementary figures and images for: Genomic analysis of carboxyl/cholinesterase genes in the silkworm Bombyx mori
Source: BMC Genomics. 2010 Jun 14;11:377. doi: 10.1186/1471-2164-11-377 (PMC3017765; doi:10.1186/1471-2164-11-377)

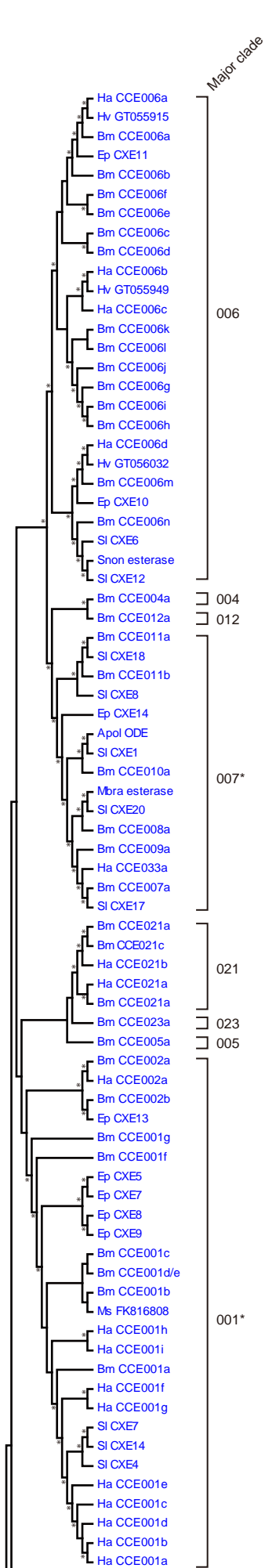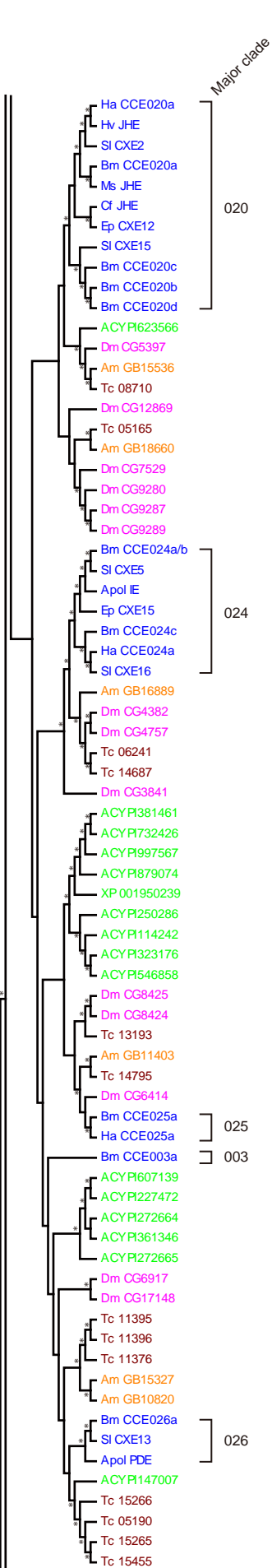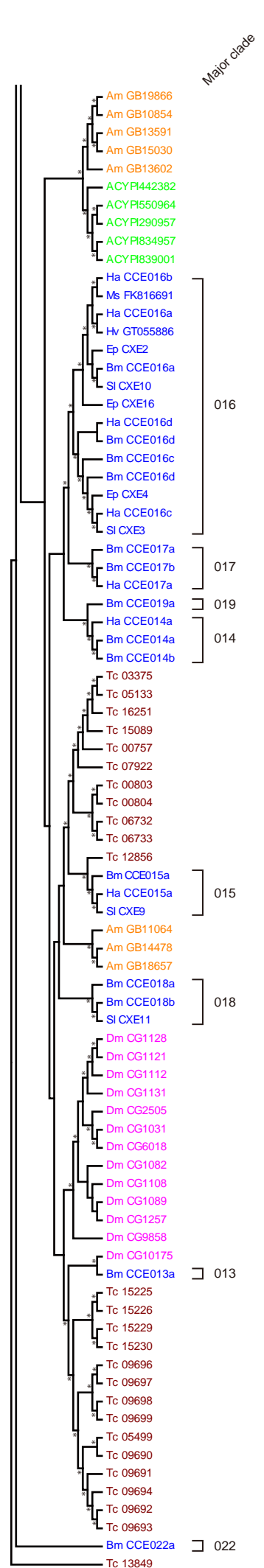

Supplement: Additional file 1 — Phylogenetic tree of insect CCEs. A phylogenetic tree containing lepidopteran, D. melanogaster, A. mellifera, T. castaneum and A. pisum CCEs. Lepidopteran CCEs are colored blue, D. melanogaster purple, A. mellifera orange, T. castaneum brown and A. pisum green. Asterisks in the cladogram indicate bootstrap values greater than 50%, and the nomenclatures of clades are according to Teese et al [23]. For simplicity, neurodevelopmental CCEs are omitted. [file 1471-2164-11-377-S1.PDF]
